# The use of herbal and dietary supplement among community-dwelling elderly in a suburban town of Malaysia

**DOI:** 10.1186/s12906-021-03287-1

**Published:** 2021-04-01

**Authors:** Mohd Shahezwan Abd Wahab, Muhammad Helmi Zaini, Aida Azlina Ali, Shariza Sahudin, Muhammad Zulfadli Mehat, Hafizah Abdul Hamid, Mohd Faiz Mustaffa, Noordin Othman, Sandra Maniam

**Affiliations:** 1 Faculty of Pharmacy, Universiti Teknologi MARA (UiTM) Cawangan Selangor, Kampus Puncak Alam, 42300 Puncak Alam, Selangor Malaysia; 2 Department of Human Anatomy, Faculty of Medicine and Health Sciences, Universiti Putra Malaysia, 43400 Serdang, Malaysia; 3 Department of Clinical and Hospital Pharmacy, College of Pharmacy, Taibah University, Al-Madinah Al-Munawwarah, 30001 Saudi Arabia; 4 Faculty of Pharmacy, PICOMS International University College, 68100 Batu Caves, Kuala Lumpur Malaysia

**Keywords:** Complementary and alternative medicine, Elderly, Herbal and dietary supplement, Malaysia

## Abstract

**Background:**

The use of herbal and dietary supplement (HDS) in health and disease management has gained global attention. HDS are generally accepted by the public and are associated with positive health behaviours. However, several reports have been documented with regards to their potential adverse effects and interaction with conventional medicines. Limited data is currently available on the use of HDS among elderly population in Malaysia. This present study aims to investigate the prevalence of and pattern of HDS use among a sample of community-dwelling elderly in a suburban town in Malaysia.

**Methods:**

A cross-sectional survey was conducted between March and May 2019 among the elderly aged ≥60 years old. The participants with the following criteria were included in the study: aged ≥60 years, residing in Puncak Alam and able to understand Malay or English language. Data were collected using a pre-validated questionnaire. All statistical analysis was conducted using IBM SPSS ver. 23.

**Results:**

Overall, 336 out of 400 elderly responded to the survey, achieving a response rate of 84%. This study observed that almost 50% of the respondents were using at least one type of HDS in the past one month of the survey. Among HDS non-users, most of them preferred to use modern medicines (62.6%, 114/182). Among the HDS users, 75.3% (116/154) were using at least one type of modern medicine (prescription or over-the-counter medicine). Multivariate analysis showed that having good to excellent perceived health (adjusted OR = 2.666, 95% CI = 1.592–4.464), having felt sick at least once in the past one month (adjusted OR = 2.500, 95% CI = 1.426–4.383), and lower body mass index (adjusted OR = 0.937, 95% CI = 0.887–0.990) were associated with HDS use. It was noted that only a small percentage of HDS users (16.2%, 25/154) had informed healthcare providers on their HDS use.

**Conclusion:**

The use of HDS is common among the elderly sampled. Hence, healthcare providers should be more vigilant in seeking information of HDS use for disease management in their elderly patients. Campaigns that provide accurate information regarding the appropriate use of HDS among the elderly are pertinent to prevent misinformation of the products.

## Background

Complementary and alternative medicine (CAM) is a broad term that covers non-mainstream practices of healthcare such as “natural products” (herbal and dietary supplements [HDS]) and “mind and body practices” [1]. The use of HDS has steadily increased over the years in many countries worldwide [2]. In a previous nationally representative survey among Malaysians, 87.3 and 88.9% of the respondents were reported to use biological-based therapies (including HDS) to maintain health and treat health problems, respectively [3]. Furthermore, HDS were commonly used by patients to manage chronic diseases [4] such as diabetes mellitus and dyslipidemia, and to maintain health [5].

The use of CAM was associated with several benefits among elderly which includes positive behaviour, which was reported among CAM users such as better confidence, empowerment, knowledge, and control relating to their health [6]. A systematic review by Bishop and colleagues showed that CAM users were more proactive in participating in treatment decisions, practice active coping strategy and were more in control of their health [7]. CAM or HDS are perceived as products that are safer with lesser side effects compared to modern medicines [7, 8]. Previous studies have associated several factors with HDS use among the elderly population such as female gender [4, 9–11], higher education [4, 9, 11–13], higher income [9, 11], ethnicity [9, 13], older age [9], use of other medications [12], and engagement in healthy lifestyles [14].

Despite the associated benefits and the perceived safety of HDS, there were concerns with the use of the products especially among the elderly [15]. HDS were shown to exert pharmacological effects due to the chemical moieties that they contain, consequently resulting in adverse effects [16]. In a report based on the 2004–2013 data from 63 hospitals in the United States (US), elderly aged 65 years or more were found to be more likely to be hospitalized due to adverse effects of dietary supplements (DS) compared to younger adults [17]. A subsequent surveillance survey reported hospitalization rates for emergency department (ED) visits due to adverse events associated with herbal medicine (HM) and DS, with data showing 10 and 12.9%, respectively [18]. It was also noted that 1.2 and 1.1% of 42,585 ED visits were due to adverse events caused by HM and DS, respectively [18].

The use of HDS has been implicated with adverse effects such as liver dysfunction [15] and nephropathy [19]. Elderlies with multiple comorbidities and usage of conventional medicines were shown to experience higher risk of adverse effects from HDS due to age-related physiological changes that affect the pharmacokinetics and pharmacodynamics of drugs consumed [20]. Case reports on HDS-drug interactions showed altered bioavailability of drugs which results in either increased risk of toxicity or reduced efficacy of conventional medicines [21]. This raises concern as elderlies are frequent users of medicines (i.e., prescriptions and over-the-counter) [22], as well as multiple types of HDS [15, 23]. In a study by Levy and colleagues, HDS-drug or HDS-HDS interactions were implicated for hospitalizations of 3.7% of all inpatients who used HDS [16].

High prevalence of HDS use among the elderly was reported in Western countries [24–27]. However, reports of HDS use among elderly in Asian countries are limited [4], and among Malaysian elderly is scarce [8]. Information gathered from previous studies conducted in other countries may not be applicable to the elderly in Malaysia. Therefore, the objectives of this present study were: (1) to investigate the prevalence of HDS use, including the types used, reasons of use, sources of obtainment, expenditure on HDS, sources of HDS information, and the disclosure to healthcare providers; (2) to examine the predictors of HDS use; and (3) to analyze the pattern of use (i.e., frequency, and purposes of use), and experience from use of each HDS utilized by a sample of community-dwelling elderly in a suburban town in Malaysia.

## Methods

### Study design and sample population

A cross-sectional study was conducted among a sample of community-dwelling elderly in Puncak Alam, a main suburban township in Kuala Selangor District, State of Selangor, Malaysia. Elderly were defined as those aged 60 years and above [28]. Elderly aged 60 years and older were recruited between March and May 2019. The participants with the following criteria were included in the study: aged ≥60 years, residing in Puncak Alam and able to understand Malay or English language. Exclusion criteria includes elderly who had cognitive or mental health problems or who refused to participate in the survey. The sample size was calculated using the Raosoft sample size calculator [29]. A sample size of 334 was recommended for a margin of error of 5%, confidence interval of 95%, and response distribution of 50% for an approximate total population of 2500 elderly in Puncak Alam [30]. This study was approved by the Research Ethics Committee of Universiti Teknologi MARA (UiTM), Malaysia (600-IRMI.5/1/6).

### Survey instrument

The questionnaire was developed based on inputs from previous studies (Appendix 1). The survey instrument was reviewed by five academic pharmacists who had experience in survey research and in studies related to CAM or HDS to assess the relevance and suitability of each survey item. The questionnaire was also tested in a small convenient sample of elderly (*n* = 10) to assess for the clarity and comprehensibility of survey items. Feedbacks obtained from the academic pharmacist reviewers and the elderly respondents in the pre-testing phase were used to improve the survey items. The pilot test showed that the survey took approximately 20–30 min.

In this study, HDS refers to natural preparations (crude) and processed products (in the form of pills, capsules, tablets, powder, and liquids) that contain plant-derived materials; or products containing dietary ingredients such as vitamins, minerals, amino acids, and substances such as enzymes, organ tissues, glands, metabolites, extracts and concentrates taken orally to treat and/or prevent diseases or maintain health in the past one month [31, 32].

The first part of the questionnaire collects important demographic details of the respondents (e.g., age, gender, race, etc.). The respondents were also asked to indicate whether they have been diagnosed with any medical condition. For those who answered “yes” were asked to state the medical conditions that they have been diagnosed with. Subsequently, the questionnaire collects details about current conventional medicine use and HDS use in the past one month. For those who answered “yes” for HDS use, they were asked about the specific type of HDS that they were using, and the frequency of use. A list of common HDS is outlined in the survey instrument. The interviewer recorded by hand the name of HDS if it is not listed in the questionnaire. The respondents were also asked about their purpose of using HDS whether to treat minor illness, to treat chronic illness, to prevent disease or to maintain health. If the respondents mentioned that they used HDS to treat minor and chronic illnesses or to prevent disease, they were further asked to state the type of disease that they intend to treat or prevent. In addition, the respondents were asked to report any positive experience (any favourable outcomes) or negative experience (any unfavourable outcomes) from HDS use. If the respondents answered that they experienced any negative effect from HDS use, they were further asked to state the negative effect. The reasons for not using HDS were obtained from the non-users whereas the users were asked about the reasons that influenced them to use the products. Respondents who were using HDS were asked if they had ever disclosed their HDS use to their healthcare providers.

### Data collection

Throughout the study period, 400 elderly were approached. The elderly respondents were selected using convenience sampling method in which potential respondents were approached at public places (e.g., supermarkets, malls, places of worship, post offices, etc.) and were asked for their interests to participate in the study. Potential respondents were screened for inclusion criteria and if eligible, were provided with a consent form to be signed. The method of interviewer-administered questionnaire was utilized as the participants were elderly who might have difficulties in self-administering the questionnaire. One research team member (MHZ) conducted all interviews to ensure a standard practice in the interview. The participation of the elderly in the study was voluntary and they were offered anonymity and confidentiality. No incentive was provided to the study participants.

### Statistical analysis

All statistical analysis was conducted using IBM SPSS ver. 23. Continuous data were presented as mean and standard deviation (SD), whereas categorical data were presented as frequency and percentage. The percentage of categorical variables was compared using the Chi-squared test or Fisher’s exact test, and continuous data were compared using the independent samples t-test. As recommended by Hosmer et al. [33], variables with a *P* value of 0.20 were analyzed using logistic regression analysis to assess the association between independent variables and the use of HDS. Odds ratios (ORs) and 95% confidence intervals (CI) were reported for the logistic regression analysis. Statistical significance was established if the *P* value was < 0.05.

## Results

### Socio-demographic characteristics of respondents

Table 1 shows the socio-demographic characteristics of the study respondents. Overall, 400 elderly were approached in the study and 336 responded to the survey, achieving a response rate of 84%. The sample met the minimum recommended sample size for this study. Those who refused to participate in the survey mentioned busyness and lack of interest as the reasons. Most of the respondents were female (224/336, 66.7%). The mean (± SD) age of the respondents was 63.57 ± 2.94, with many of them falling in the 60–65 age group (228/336, 67.9%).

**Table 1:** Socio-demographic characteristics of study respondents and comparison of the socio-demographic characteristics among HDS users and non-users

Characteristics	HDS users (***n*** = 154)	HDS non-users (***n*** = 182)	***P***^***a***^	All
Gender				
Male	43 (27.9)	69 (37.9)	0.053	112 (33.3)
Female	111 (72.1)	113 (62.1)		224 (66.7)
Age (mean ± SD)	63.45 ± 2.99	63.66 ± 2.89	0.525^b^	63.57 ± 2.94
Range	60–71	60–77		60–77
Age group				
60–65 years	105 (68.2)	123 (67.6)	0.907	228 (67.9)
> 65 years	49 (31.8)	59 (32.4)		108 (32.1)
Race				
Malay	98 (63.6)	112 (61.5)	0.205	210 (62.5)
Chinese	29 (18.8)	32 (17.6)		61 (18.2)
Indian	15 (9.7)	30 (16.5)		45 (13.4)
Others	12 (7.8)	8 (4.4)		20 (6)
Having a spouse				
Yes	113 (73.4)	127 (69.8)	0.467	240 (71.4)
No (single / divorcee / widow / widower)	41 (26.6)	55 (30.2)		96 (28.6)
Living arrangement				
Living alone	24 (15.6)	29 (15.9)	0.930	53 (15.8)
Living with other family members / caretakers	130 (84.4)	153 (84.1)		283 (84.2)
Previous education				
Secondary level and lower	101 (65.6)	104 (57.1)	0.118	205 (61)
Tertiary level	53 (34.4)	78 (42.9)		131 (39)
Employment status				
Active	106 (68.8)	138 (75.8)	0.152	244 (72.6)
Retired	48 (31.2)	44 (24.2)		92 (27.4)
Monthly income				
Less than RM 1000	105 (68.2)	92 (50.5)	0.001	197 (58.6)
More than RM 1000	49 (31.8)	90 (49.5)		139 (41.4)
Diagnosed with a medical condition				
Yes	76 (49.4)	79 (43.4)	0.276	155 (46.1)
No	78 (50.6)	103 (56.6)		181 (53.9)
Using at least one prescription medicine				
Yes	59 (38.3)	72 (39.6)	0.815	131 (39)
No	95 (61.7)	110 (60.4)		205 (61)
Using at least one OTC medicine				
Yes	94 (61)	105 (57.7)	0.534	199 (59.2)
No	60 (39)	77 (42.3)		137 (40.8)
Using at least one type of medicine (prescription or OTC)				
Yes	116 (75.3)	142 (78)	0.560	258 (76.8)
No	38 (24.7)	40 (22)		78 (23.2)
Perceived health status				
Very poor - Fair	52 (33.8)	105 (57.7)	< 0.001	157 (46.7)
Good - Excellent	102 (66.2)	77 (42.3)		179 (53.3)
Felt sick in the past month				
Never	36 (23.4)	69 (37.9)	0.004	105 (31.3)
At least once	118 (76.6)	113 (62.1)		231 (68.8)
Physical exercise at least 20 min 3 times a week				
Yes	104 (67.5)	97 (53.3)	0.008	201 (59.8)
No	50 (32.5)	85 (46.7)		135 (40.2)
Estimated number of fruit and vegetable servings daily				
≤ 2	101 (65.6)	128 (70.3)	0.352	229 (68.2)
≥ 3	53 (34.4)	54 (29.7)		107 (31.8)
Number of meals skipped in the past week				
0–3	86 (55.8)	87 (47.8)	0.223	173 (51.5)
4–6	47 (30.5)	59 (32.4)		106 (31.5)
More than 6	21 (13.6)	36 (19.8)		57 (17)
Smoking history				
Yes	40 (26)	33 (18.1)	0.086	73 (21.7)
No	114 (74)	149 (81.9)		263 (78.3)
Body mass index^c^ (mean ± SD)	27.24 ± 3.62	29.07 ± 5.17	< 0.001^b^	

OTC, over-the-counter; SD, standard deviation

^a^ Chi-squared test used unless stated otherwise

^b^ Independent samples t-test used

^c^ Calculated using self-reported weight and height (formula: BMI weight (kg)/[height (m)]2

Among the elderly sample, 46.1% (155/336) was diagnosed with a medical condition. About 77% (258/336) of the respondents were using at least one type of medicine (prescription or over-the-counter [OTC]) at the point of survey. Approximately 50% (179/336) of the respondents perceived their health as “good – excellent”. However, about 69% (231/336) of the elderly reported that they had felt sick for at least once in the past one month. Self-reported underlying conditions of the respondents are outlined in Table 2. The most common medical conditions among the elderly respondents were hypertension (64/336, 19%), diabetes mellitus (43/336, 12.8%), and hypercholesterolemia (21/336, 6.3%). The use of HDS was significantly associated with elderly having hypertension, osteoarthritis, sinusitis, and tonsillitis.

**Table 2:** Underlying medical conditions among respondents

Underlying medical condition ^**a**^	HDS user ***n*** = 154	Non HDS user ***n*** = 182	***P***^***a***^	Total ***n*** = 336
Hypertension	42 (27.3)	22 (12.1)	< 0.001	64 (19)
Diabetes mellitus	11 (7.1)	32 (17.6)	0.004	43 (12.8)
Hypercholesterolemia	12 (7.8)	9 (4.9)	0.283	21 (6.3)
Osteoarthritis	10 (6.5)	0 (0.0)	< 0.001^b^	10 (3)
Peptic ulcer disease	0 (0.0)	10 (5.5)	0.002^b^	10 (3)
Eczema	0 (0.0)	7 (3.8)	0.017^b^	7 (2.1)
Sinusitis	7 (4.5)	0 (0.0)	0.004^b^	7 (2.1)
Asthma	1 (0.6)	4 (2.2)	0.380^b^	5 (1.5)
Tonsillitis	5 (3.2)	0 (0.0)	0.020^b^	5 (1.5)
Anemia	0 (0.0)	4 (2.2)	0.128^b^	4 (1.2)
Scoliosis	3 (1.6)	0 (0.0)	0.253^b^	3 (0.9)
Carpal tunnel syndrome	0 (0.0)	2 (1.1)	0.502	2 (0.6)
Gout	0 (0.0)	2 (1.1)	0.502^b^	2 (0.6)
Benign prostatic hyperplasia	0 (0.0)	1 (0.5)	1.000^b^	1 (0.3)
Dermatitis	0 (0.0)	1 (0.5)	1.000^b^	1 (0.3)

^a^ Chi-squared test used unless stated otherwise

^b^ Fisher’s exact test used

### Factors associated with HDS use

Overall, 45.8% (154/336) of respondents were using at least one type of HDS at the point of survey. Table 1 shows the comparison of the socio-demographic characteristics of the HDS users and non-users. The univariate analysis showed that monthly income, perceived health status, history of sickness in the past one month, physical activities, and body mass index were associated with HDS use (Table 1). Multivariate analysis showed that having good – excellent perceived health (adjusted OR = 2.666, 95% CI = 1.592–4.464), a history of sickness in the past one month (adjusted OR = 2.500, 95% CI = 1.426–4.383), having lower body mass index (adjusted OR = 0.937, 95% CI = 0.887–0.990) were predictors of HDS use (Table 3).

**Table 3:** Factors associated with the use of HDS by logistic regression analysis (n = 336)

Variables	Univariate Crude OR (95% CI)	***P***	Multivariate Adjusted OR (95% CI)	***P***
Female sex	1.576 (0.993–2.502)	0.054	1.870 (0.930–3.761)	0.079
Lower education	1.429 (0.917–2.227)	0.114	0.759 (0.406–1.409)	0.379
Inactive employment	1.420 (0.878–2.298)	0.153	0.778 (0.385–1.575)	0.486
Low monthly income	2.096 (1.341–3.276)	0.001	1.956 (0.966–3.962)	0.062
Perceived health: good – excellent	2.675 (1.714–4.174)	< 0.001	2.666 (1.592–4.464)	< 0.001
Felt sick at least once	2.001 (1.240–3.230)	0.004	2.500 (1.426–4.383)	0.001
Physical exercise at least 20 min 3 times a week	1.823 (1.167–2.846)	0.008	1.291 (0.749–2.224)	0.357
Had smoking history	1.584 (0.941–2.669)	0.084	1.994 (0.959–4.105)	0.065
Body mass index^a^	0.912 (0.866–0.959)	< 0.001	0.937 (0.887–0.990)	0.020

^a^ Calculated using self-reported weight and height (formula: BMI = weight (kg)/[height (m)]^2^

Among HDS non-users (182/336, 54.2%), the most common reasons for not using HDS include the preference for modern medicine (114/182, 62.6%), having no interest in using HDS (108/182, 59.3%), and the perception that HDS was too expensive (69/182, 37.9%) (Table 4).

**Table 4:** Non-users’ reasons for not using HDS (*n* = 182)

Reason for not using HDS ^**a**^	Frequency (%)
I prefer to use modern medicine	114 (62.6)
I am not interested in using HDS	108 (59.3)
Too expensive	69 (37.9)
I am not familiar about the health benefits of HDS	63 (34.6)
HDS will not work to resolve my symptoms / to maintain health	60 (33)
HDS is not safe	45 (24.7)
HDS is unscientific	36 (19.8)
Negative experience from previous use	29 (15.9)
Not recommended by healthcare practitioners	25 (13.7)
Difficult to obtain	21 (11.5)
Not recommended by family and friends	18 (9.9)
Not necessary	9 (4.9)

^a^ Respondents can provide more than one response and therefore responses do not add up to 100%

*HDS* herbal and dietary supplement

### Details about HDS use among users

Table 5 shows the details of HDS use among the users. At the point of survey, 206 HDS products were used by 154 elderly. The average number (± SD) of HDS used by the users was 1.34 ± 0.55 (range = 1–3). Of the users, 69.5% (107/154) were using one type of HDS whereas 30.5% (47/154) were using two or three types of HDS. The most common types of HDS used by users were vitamin C (60/154, 39%), honey (35/154, 22.7%), fish oil (15/154, 9.7%), glucosamine (14/154, 9.1%), traditional herbal preparation i.e. *jamu / makjun* (12/154, 7.8%), and vitamin B complex (12/154, 7.8%).

**Table 5:** HDS use among users (*n* = 154)

HDS use	Frequency (%)
Number of HDS use	
Using one type of HDS	107 (69.5)
Using two types of HDS	39 (25.3)
Using three types of HDS	8 (5.2)
Classification of HDS use	
Using herbal medicine only	21(13.6)
Using dietary supplement only	116 (75.3)
Using both herbal medicine and dietary supplements	17 (11)
Average number of HDS used by respondents (mean ± SD)	1.34 ± 0.55
Range	1–3
Type of HDS currently using^a^	
Vitamin C	60 (39)
Honey	35 (22.7)
Fish oil	15 (9.7)
Glucosamine	14 (9.1)
Traditional herbal preparation (*jamu / makjun*)	12 (7.8)
Vitamin B complex	12 (7.8)
Vitamin B12	11 (7.1)
Commercial herbal juice	9 (5.8)
Multivitamin	7 (4.5)
Tongkat Ali (*Eurycoma longifolia*)	6 (3.9)
Apple cider vinegar	5 (3.2)
Colostrum	4 (2.6)
Homemade herbal juice	4 (2.6)
Ginger (crude)	3 (1.9)
Turmeric capsule	3 (1.9)
Zinc	3 (1.9)
Apricot seed	2 (1.3)
Vitamin E	1 (0.6)
Reason for HDS use^a^	
Easy to obtain	139 (90.3)
Affordable	132 (85.7)
Recommendation from family and friends	127 (82.5)
I want to try HDS	122 (79.2)
HDS is effective	98 (63.6)
My problem is not serious enough to use modern medicine	84 (54.5)
Safe	79 (51.3)
I prefer to use HDS rather than using modern medicine	68 (44.2)
I am familiar with the health benefits of HDS	48 (31.2)
Recommendation from healthcare practitioners	33 (21.4)
Disclosure to healthcare professionals	
Yes	25 (16.2)
No	133 (86.4)
Source of HDS^a^	
Pharmacy	86 (55.8)
Family and friends	51 (33.1)
Supermarket	34 (22.1)
Grocery store	12 (7.8)
Traditional medicine outlet	5 (3.2)
Night market	4 (2.6)
Online	4 (2.6)
Health store / organic store	0 (0)
Spending on HDS	
Less than RM 100	125 (81.2)
RM 100 – RM 200	14 (9.1)
More than RM 200	4 (2.6)
Unspecified	15 (9.7)
Source of information about HDS^a^	
Family and friends	142 (92.2)
Internet	59 (38.3)
Television	56 (36.4)
Newspapers	41 (26.6)
Medical practitioners	40 (26)
Pharmacists	36 (23.4)
Books	13 (8.4)
Radio	9 (5.8)

*HDS* herbal and dietary supplement

^a^ Respondents can provide more than one response and therefore responses do not add up to 100%

The common reasons that motivated users to use HDS include easy access to the products (139/154, 90.3%), affordable price (132/154, 85.7%), and recommendation from family and friends (127/154, 82.5%). Among all HDS users, only a small percentage of them (25/154, 16.2%) informed their HDS use to their healthcare providers. The top three sources of HDS cited by users were pharmacies (86/154, 55.8%), followed by family and friends (51/154, 33.1%), and supermarkets (34/154, 22.1%). The majority of HDS users (125/154, 81.2%) spent less than RM 100 a month for HDS products. HDS users mostly obtained information about HDS from family and friends (142/154, 92.2%), the Internet (59/154, 38.3%), and television (56/154, 36.4%).

### Pattern and experience of use among HDS users

Of all 206 HDS products used by the users, the majority used the products daily (125/206, 60.7%) (Table 6). The products were mainly used to maintain health (173/206, 84%) or prevent diseases (86/206, 41.7%). About 25% (51/206) of these products were used to treat minor illnesses whereas 14.6% (30/206) were used to treat chronic diseases. Most of the products (194/206, 94.2%) were reported to produce positive results. Only a small percentage of the products (20/206, 9.7%) were reported to produce adverse effects. The common adverse effects reported included weight gain (*n* = 5), stomachache (*n* = 4), and frequent urination (n = 4) (see footnote in Table 6). Table 7 summarizes the reported use of HDS among the users.

**Table 6:** Pattern and experience of HDS use among users

Name of products	Frequency of use			Purpose for using^**a**^				Experience positive effect^**b**^		Experience negative effect^**b**^	
	Once a week	Few times a week	Daily	Treat minor illness	Treat chronic illness	Prevent disease	Maintain health	Yes	No	Yes	No
Vitamin C (*n* = 60)	3 (5)	25 (41.7)	32 (53.3)	20 (33.3)	3 (5)	36 (60)	49 (81.7)	55 (91.7)	5 (8.3)	8 (13.3)^c^	52 (86.7)
Honey (*n* = 35)	1 (2.9)	18 (51.4)	16 (45.7)	21 (60)	–	12 (34.3)	30 (85.7)	35 (100)	–	1 (2.9)^d^	34 (97.1)
Fish oil (*n* = 15)	–	4 (26.7)	11 (73.3)	–	5 (33.3)	12 (80)	15 (100)	12 (80)	3 (20)	1 (6.7)^e^	14 (93.3)
Glucosamine (*n* = 14)	–	–	14 (100)	–	14 (100)	–	–	14 (100)	–	–	14 (100)
Traditional herbal preparation e.g. *jamu / makjun* (*n* = 12)	–	7 (58.3)	5 (41.7)	–	–	5 (41.7)	12 (100)	12 (100)	–	1 (8.3)^f^	11 (91.7)
Vitamin B complex (*n* = 12)	–	4 (33.3)	8 (66.7)	–	–	3 (25)	12 (100)	12 (100)	–	1 (8.3)^g^	11 (91.7)
Vitamin B12 (*n* = 11)	–	1 (9.1)	10 (90.9)	–	–	5 (45.5)	11 (100)	11 (100)	–	–	11 (100)
Commercial herbal juice (*n* = 9)	–	3 (33.3)	6 (66.7)	5 (55.6)	2 (22.2)	2 (22.2)	9 (100)	7 (77.8)	2 (22.2)	2 (22.2)^h^	7 (77.8)
Multivitamin (*n* = 7)	–	2 (28.6)	5 (71.4)	–	–	2 (28.6)	7 (100)	7 (100)	–	–	7 (100)
Tongkat Ali / *Eurycoma longifolia* (*n* = 6)	–	3 (50)	3 (50)	–	–	–	6 (100)	6 (100)	–	–	6 (100)
Apple cider vinegar (*n* = 5)	–	1 (20)	4 (80)	–	2 (40)	1 (20)	4 (80)	5 (100)	–	–	5 (100)
Colostrum (*n* = 4)	–	2 (50)	2 (50)	–	4 (100)	1 (25)	4 (100)	2 (50)	2 (50)	–	4 (100)
Homemade herbal juice (*n* = 4)	–	–	4 (100)	–	–	1 (25)	4 (100)	4 (100)	–	3 (75)^i^	1 (25)
Crude ginger (*n* = 3)	2 (66.7)	1 (33.3)	–	2 (66.7)	–	1 (33.3)	1 (33.3)	3 (100)	–	1 (33.3)^j^	2 (66.7)
Turmeric capsule (*n* = 3)	–	–	3 (100)	–	–	3 (100)	3 (100)	3 (100)	–	–	3 (100)
Zinc (*n* = 3)	2 (66.7)	1 (33.3)	–	3 (100)	–	–	3 (100)	3 (100)	–	2 (66.7)^k^	1 (33.3)
Apricot seed (*n* = 2)	–	1 (50)	1 (50)	–	–	2 (100)	2 (100)	2 (100)	–	–	2 (100)
Vitamin E (*n* = 1)	–	–	1 (100)	–	–	–	1 (100)	1 (100)	–	–	1 (100)
Total products used (*n* = 206)	8 (3.9)	73 (35.4)	125 (60.7)	51 (24.8)	30 (14.6)	86 (41.7)	173 (84)	194 (94.2)	12 (5.8)	20 (9.7)	186 (90.3)

^a^ Respondents can provide more than one response and therefore responses do not add up to 100%. ^b^ Self-reported; positive experience refers to any favourable outcomes experienced by the users; negative experience refers to any unfavourable outcomes experienced by the users

^c^ Stomachache (*n* = 4); increased appetite (*n* = 1); weight gain (*n* = 3)

^d^ Increased appetite (*n* = 1)

^e^ Increased appetite (*n* = 1)

^f^ Flatulence (*n* = 1)

^g^ Diarrhea (*n* = 1)

^h^ Frequent urination (*n* = 2)

^i^ Frequent urination (*n* = 2); diarrhea (*n* = 1)

^j^ Flatulence (*n* = 1)

^k^ Weight gain (*n* = 2)

**Table 7:** Reported use of HDS among users

	Treat minor illness	Treat chronic illness	Prevent disease
Vitamin C	Cold and Flu (*n* = 7)	Migraine (*n* = 3)	Cold and Flu (*n* = 13)
	Fever (*n* = 6)		Hypertension (*n* = 6)
	Skin itchiness (*n* = 3)		Fever (*n* = 5)
	Constipation (*n* = 3)		Diabetes mellitus (*n* = 4)
	Cough (*n* = 1)		Cancer (*n* = 3)
			Skin itchiness (*n* = 3)
			Stroke (*n* = 1)
			Cough (*n* = 1)
Honey	Cough (*n* = 11)	–	Diabetes mellitus (*n* = 4)
	Fever (*n* = 4)		Hypertension (*n* = 2)
	Sore throat (*n* = 4)		Cold and Flu (*n* = 2)
	Cold and Flu (*n* = 1)		Cancer (*n* = 1)
	Oral ulcer (*n* = 1)		Cough (*n* = 1)
			Fever (*n* = 1)
			Stroke (*n* = 1)
Fish oil	–	Hypertension (*n* = 4)	Hypertension (*n* = 9)
		Eye disease (*n* = 1)	Heart disease (*n* = 1)
			Eye disease (*n* = 1)
			Cancer (*n* = 1)
Traditional herbal preparation (*jamu* / *makjun*)	–	–	Obesity (*n* = 4)
			Flatulence (*n* = 1)
Glucosamine	–	Osteoarthritis (*n* = 14)	–
Vitamin B complex	–	–	Alzheimer’s disease (*n* = 1)
			Heart disease (*n* = 1)
			Indigestion (*n* = 1)
Vitamin B12	–	–	Heart disease (*n* = 3)
			Fever (*n* = 1)
			Osteoporosis (*n* = 1)
Commercial herbal juice	Body pain (*n* = 5)	Osteoarthritis (*n* = 2)	Hypercholesterolemia (*n* = 2)
Multivitamin	–	–	Osteoporosis (*n* = 2)
Apple cider vinegar	–	Diabetes mellitus (*n* = 2)	Diabetes mellitus (*n* = 1)
Colostrum	–	Osteoarthritis (*n* = 3) Migraine (*n* = 1)	Osteoporosis (*n* = 1)
Homemade herbal juice	–	–	Bloating (*n* = 1)
Ginger (crude)	Flatulence (*n* = 2)	–	Bloating (*n* = 1)
Turmeric capsule	–	–	Cancer (*n* = 3)
Zinc	Cold and flu (*n* = 3)	–	–
Apricot seed	–	–	Cancer (*n* = 2)

## Discussion

The finding from this study reports the common use of HDS among Malaysian elderly with the prevalence of 45.8%. It was also noted that the use of at least one type of DS and one type of HM has the prevalence of 39.5 and 11.3%, respectively. To our knowledge, this is the first study conducted that focused on HDS use among a sample of community-dwelling elderly in a suburban town in Malaysia. Previous studies have been conducted in urban cities [8, 34, 35] and rural areas [35] with the prevalence of HDS use of 43–56.9%. The prevalence of HDS use in this study was comparatively similar to few studies conducted in Ghana [12], Australia [36] and US [37]. The prevalence was comparatively lower to that reported in several other US studies [24, 25, 38] and in a study in Germany [26]. Factors associated with HDS use among the elderly that have been reported in previous studies included female gender [4, 9–11], higher education [4, 9, 11–13], higher income [9, 11], ethnicity [9, 13], older age [9], use of other medications [12], and engagement in healthy lifestyles [14]. This present study showed that elderly who perceived their health as “good – excellent”, who had a history of sickness in the past one month or who had lower body mass index were more likely to use HDS. The discrepancies in the prevalence and factors associated with HDS use among elderly between studies could be due to various factors such as the study design, recall period, type of HDS included age group of study population, settings and location.

Similar to previous studies, HDS users in the present study were more health conscious, which was evident by the higher engagement in weekly physical activities [14, 39, 40]. However the non-significant finding on smoking history (i.e., current or former smoker) among HDS users in this study was contradictory from several other studies that showed CAM or HDS users were more likely to refrain from smoking [9, 14]. Past reports on former smokers in a large cohort of community-dwelling elderly in the Gingko Evaluation Memory (GEM) study showed that this group was associated with the use of specific vitamin and mineral supplement [41]. Current or former smoker was also associated with HDS use among young adults in the US [42] and was most likely to use HDS in Danish women [43]. It is possible that those with smoking history are more health conscious and may consider using HDS to improve their health status.

Despite the health-attentive behaviour observed in elderly HDS users, they were more likely to fall sick at least once in the past month. This finding was in agreement with other studies where the elderly were reported to experience higher number of physically and mentally unhealthy days among HDS users despite being more health conscious [14]. Similarly, reports of arthritis and/or depression/anxiety, and frequent doctor visits were noted in elderly CAM users who are engaged in exercises [39].

Our results suggest that HDS consumption is influenced by high motivation to maintain health and the desire to solve health problems as approximately 80% of the of the HDS users “want to try HDS” (see Table 5). Additionally, it was also observed that the majority of HDS products (84%) were used to maintain health and about 42% of the products were used to prevent diseases. These findings are noteworthy given the public health imperative that more people become proactive and motivated to take an active role in their health management.

The consumption of HDS may also be attributed to the marketing of the products via the media or word of mouth. This study reports the HDS products were either recommended (82.5%) or product information were received (92.2%) from family and friends. Media such as the internet, television, and newspapers were also cited as common sources of HDS information among the users. There is a concern that information obtained from family and friends, and popular media may not be reliable, and may be anecdotal in nature and misleading [44]. Of note, many of the reported purposes for HDS use either for prevention of diseases or treatment of common ailments and chronic diseases were either unproven or not supported by existing evidence (e.g., the use of apricot seed, honey, turmeric, and fish oil to prevent cancer; apple cider vinegar, vitamin C and honey to treat / prevent diabetes mellitus; colostrum supplement to treat migraine and osteoarthritis) (see Table 7). This finding suggests that elderly who used HDS may not be well-informed or have been misled about the products. It is worth noting that although approximately 56% of HDS users obtained their HDS products from the pharmacies, only about 23% reported “pharmacists” as their source of information for their use of HDS. This finding suggests that communication about HDS among pharmacists and HDS customers at the pharmacy is minimal. Factors influencing the HDS users not to consider pharmacists as their preferred source of HDS information warrants further investigation.

The present study showed that 38.3 and 61% of HDS users were using prescription and OTC medicines, respectively. The prevalence of use of at least one type of medicine (prescription or OTC) was high at 75.3%. Our results emulate findings from a previous study that reported 68% of elderly were using OTC and HDS in addition to their prescribed medicines [10]. In another study among elderly patients with depression and/or dementia, 75% of the HDS users were also noted to be taking other medications including psychotropic and psychoactive drugs [45]. Furthermore, in the GEM study cohort, 90% of elderly who used HDS were also found to be using prescription medicine [24]. Concurrent use of HDS and conventional medicines should be a cause for concern due to the potential risk of HDS-drug interactions. The information on the clinical importance of HDS-drug interactions is limited. However, these interactions may potentially result in altered bioavailability or efficacy, or enhanced toxicity of conventional medicines [20].

A concern arose from this study as well as other reported studies [4, 46–49] is the non-disclosure of HDS consumption by users to their healthcare providers. A study by Mitha et al. also reported the absence of consultation with healthcare professional before using the treatment modalities among elderly CAM users [8]. This is an issue as without such disclosure, healthcare providers may not have the opportunity to assess the appropriateness of HDS use, detect HDS-related problems (e.g., toxicity, interactions, etc.) or provide professional advice regarding its use. Based on previous surveys that included HDS as part of CAM, the common reasons for non-disclosure of CAM use to healthcare providers include the lack of time to discuss about CAM [50], “healthcare providers did not ask” [14, 50, 51] and being unsure about the necessity or the importance of disclosure [14, 50].

Despite the safety concerns related to HDS use, our study showed that the HDS users were generally satisfied with the products. From 206 HDS products used in this study, 94.2% were claimed to produce positive effects which is in agreement with previously reported studies [14, 52, 53]. In a study conducted among elderly Japanese and American outpatients, CAM was even perceived as more effective than the treatment prescribed by physicians [54]. Similarly, in a survey among a sample of elderly in Malaysia, 55.1% of the respondents believed that CAM were more effective than modern medicines [8]. Moreover, only about 10% of HDS products in the study were implicated with negative experience. There are at least two possible explanations for these findings. First, HDS use may promote overall health (rather than focusing on illness), resulting in positive perceived outcomes among the users. Secondly it is also possible that HDS users are less wary about the potential adverse effects of HDS thus neglecting possible reactions.

High satisfaction from HDS use may result in abandoning prescribed medicine among HDS users [55]. Of note, in this present study, HDS was used to treat chronic diseases such as hypertension and diabetes mellitus. However, this study did not capture the rate of non-compliance of HDS users to their prescribed medicines. Therefore, it is unknown whether the use of HDS affects medications adherence. Hence, it is important for healthcare providers to remain cognizant of HDS use among the elderly so that the use of proven conventional therapies is not delayed or abandoned.

High satisfaction from HDS use among the respondents implies a general acceptance of the products among the elderly population [2]. The findings from this study suggest the use of HDS among the elderly may continue to be widespread which is currently seen in other developed countries [24, 26, 27]. The widespread use of HDS reported in our study warrants the on-going campaign to educate the public about HDS benefits and risks. There is also a pressing need for healthcare providers to continue learning about HDS and to integrate HDS in their professional practices [56].

### Limitations

The present study has several limitations. First, being a cross-sectional study, it only provides a snapshot of pattern of HDS use among the sample of elderly at the point of survey. As demonstrated in the study by Goh and colleagues, the pattern of HDS use among elderly may change (i.e., increased or decreased) over time [57]. Furthermore, the use of HDS and other data collected (including medical conditions and medication use) were self-reported. It is likely that respondents under- or over-estimate their use of HDS and other information. In addition, the study did not assess the dosage form, and the amount of intake or dose of each HDS the users were taking. Additionally, although we identified that 75.3% of elderly were using at least one type of medicine (prescription or OTC) with HDS, we did not assess the presence of HDS-drug interactions. Lastly, although the study met the recommendation for the minimum sample size, the sample of elderly recruited was from only one suburban town. The recruitment of elderly sample from only one geographical region limits the generalization of our study findings. The use of HDS may be different if similar study is conducted in metropolitan or rural areas. It is also possible that those who responded to the survey might be more interested in HDS (although were not using it), predisposing the results of this study to sampling bias. Future studies may be carried out in a bigger population of elderly individuals and from multiple sites.

## Conclusions

HDS use was common among the elderly sample in this study. The elderly in general had positive experience with using the products. Even so, several concerns about HDS use in the study sample were noted. Our study findings showed that HDS were being concomitantly used with prescribed or OTC medicines. The rate of disclosure on HDS use to the healthcare providers was low. It was observed that easy access to HDS products, its affordability and recommendations from family and friends may have influenced the elderly to use HDS. HDS users were also noted to use unscientific source of information on HDS. Therefore, healthcare providers especially the pharmacists should be vigilant of the use of HDS in the elderly and actively play bigger roles in ensuring the safe and appropriate use of HDS in this population. Given that the elderly generally did not disclose their HDS use, this information should be solicited by healthcare providers while providing patient care services. Additionally, healthcare providers such doctors and pharmacists must equip themselves with adequate knowledge on HDS so that they can provide advice on safe and appropriate use of HDS to the consumers.

## Data Availability

The datasets used and/or analyzed during the current study are available from the corresponding author on reasonable request.

## Abbreviations

CAM: Complementary and alternative medicine
CI: Confidence intervals
DS: Dietary supplement
ED: Emergency department
GEM: Gingko Evaluation Memory
HDS: Herbal and dietary supplement
HM: Herbal medicine
OR: Odds ratio
OTC: Over-the-counter
SD: Standard deviation
US: United States
